# Effects of Two Tempering Treatments at Different Temperatures on Microstructure and Room/High-Temperature Wear Resistance of H13 Steel

**DOI:** 10.3390/ma19122585

**Published:** 2026-06-16

**Authors:** Weiwei Song, Yongbin Liu, Shan Tang, Mengyuan Dai, Zhijun Wu

**Affiliations:** 1 School of Electrical Engineering and Automation, Anhui University, Hefei 230039, China; sww_hsu@163.com; 2 Anhui Tunxi High Pressure Valve Co., Ltd., Huangshan 245061, China; wzj_tunfa@163.com; 3 School of Mechanical and Electrical Engineering, Huangshan University, Huangshan 245041, China; 4 School of Intelligent Manufacturing, Anhui University of Science and Technology, Chuzhou 233000, China; ts_406@163.com

**Keywords:** H13 steel, double tempering, microstructure, microhardness, wear resistance

## Abstract

As a typical Cr-Mo-V series hot work die steel, H13 steel is widely used in hot extrusion dies under harsh service conditions. Tempering is a vital post-quenching process for regulating microstructural evolution and comprehensive mechanical properties. Since relevant systematic comparative studies remain insufficient, industrial-grade H13 steel was adopted in this work. Specimens were quenched at 1000 °C, followed by single tempering at 520 °C and double tempering at 580 °C. Their microstructure, microhardness, and wear resistance at 25 °C and 580 °C were characterized, and the underlying mechanisms were analyzed. The results show that single tempering at 520 °C produces tempered martensite and finely dispersed carbides with secondary hardening behavior. Its microhardness reaches 590.83 HV, resulting in the best wear resistance at both room and high temperatures. Double tempering at 580 °C causes carbide coarsening, and the microhardness slightly declines to 580.60 HV. Although toughness is enhanced and residual stress is fully released, wear resistance deteriorates. This study optimizes the tempering parameters for H13 steel, provides technical support for die production, and offers theoretical guidance for the technical upgrading of the hot work die steel industry.

## 1. Introduction

H13 steel is widely used under complex working conditions, such as in hot extrusion dies, owing to its excellent mechanical properties. Its corresponding international standard grade is 40CrMoV5, and the domestic grade in China is 4Cr5MoSiV1, which belongs to a typical Cr-Mo-V series hot work die steel. The reasonable proportion of alloying elements such as Cr, Mo, and V in its chemical composition endows it with good hardenability, high-temperature strength, wear resistance, and thermal fatigue performance [1]. Dies made of H13 steel need to withstand complex service conditions for a long time, which are mainly reflected in two aspects: first, they undergo alternating cycles of high and low temperatures, which are likely to produce periodic thermal stress; second, they need to bear mechanical impact, extrusion, and surface friction and wear from workpieces, which can lead to failure modes such as wear, deformation, and cracking, among which wear failure accounts for more than 60% of total die failure [2]. Studies have shown that an unreasonable heat treatment process will lead to severely inadequate performance of H13 steel and significantly shorten its service life; therefore, the heat treatment process of H13 steel is crucial. The heat treatment process of H13 steel mainly includes quenching and subsequent tempering treatment, among which tempering treatment is an indispensable key process after quenching that directly determines the microstructure and related mechanical properties of H13 steel [3].

After quenching, H13 steel readily forms a martensitic and retained-austenite structure. Martensite can significantly improve the hardness and strength of the steel, while retained austenite is an undesirable phase that adversely affects material hardness and performance. Meanwhile, quenching generates substantial internal stress in the steel, which further decreases toughness and increases brittleness. Thus, quenched H13 steel is unable to be directly applied in engineering practice [4]. Tempering treatment is an effective method to solve this problem. Through tempering, the martensite in quenched H13 steel can be decomposed, fine and dispersed carbides can be precipitated, the internal stress generated during quenching can be released, and its microstructure can be re-regulated, thereby ensuring that H13 steel has high hardness and strength while maintaining its toughness and wear resistance [5]. At present, single and double tempering processes are widely used in industrial production to optimize the comprehensive performance of H13 steel.

Numerous published works have elaborated on the general relationship between tempering regimes, microstructure and basic mechanical properties of H13 steel, and plenty of studies have discussed how tempering temperature and cycles affect martensite transformation, carbide precipitation and macroscopic hardness.

Nevertheless, most existing studies focus on conventional mechanical properties at room temperature, and few reports quantitatively compare the differences in microstructural evolution and high-temperature wear behavior between single and double tempering. Moreover, the correlation between tempering parameters, characteristic microstructure, and service performance under actual high-temperature working conditions has not been systematically clarified, representing the specific research gap that this work aims to address.

Existing studies have shown that double tempering can further eliminate quenching internal stress, promote the transformation of retained austenite into martensite in H13 steel, and ensure that the hardness does not decrease while improving the toughness of the steel. At the same time, the fine carbides precipitated from tempered martensite during tempering can cause secondary hardening of H13 steel, so that the hardness of the workpiece can be restored to the quenched level [6].

Many prior investigations have confirmed that the size, morphology and distribution of carbides, as well as the matrix structure of tempered martensite, are core factors governing the wear resistance of die steel. Most scholars believe that uniformly distributed fine carbides can hinder abrasive wear, while coarse carbides will peel off under friction and accelerate material loss. However, relevant discussions targeting H13 steel under different tempering cycles and high-temperature service environments are still superficial.

In addition, multiple tempering is expected to promote the precipitation of more fine and dispersed strengthening phases in H13 steel, thereby improving its overall performance, but some studies have pointed out that an excessive number of tempering cycles will lead to carbide coarsening, which in turn weakens the hardness and wear resistance of H13 steel [7]. In practical engineering applications, the service temperature of H13 steel dies is mostly in the range of room temperature to 600 °C, and the surface wear resistance of die steel at high temperatures directly affects the service performance of the die. At present, comparative research on the effect of double tempering on the high-temperature wear resistance of H13 steel is relatively scarce, so carrying out research in this direction has important engineering application value.

To fill the above research gaps, clarify the influence patterns of single and double tempering on the microstructure, hardness, and room/high-temperature wear resistance of H13 steel, and reveal the internal mechanism, this study used H13 steel as the test material.

It should be noted that friction and wear are complex systemic surface behaviors, which are not only dominated by material microstructure and hardness, but also closely affected by surface state, thermal softening effect and interfacial contact conditions under high temperature.

On the basis of the same quenching process, different tempering temperatures were set to perform single and double tempering, respectively. The various mechanical properties of H13 steel under the two tempering methods were systematically compared, and the tempering process parameters of H13 steel were optimized, thereby providing technical support for the production and manufacturing of H13 steel dies and offering theoretical and practical guidance for the subsequent technological upgrading of the hot work die steel industry.

Against the aforementioned research gaps and insufficient theoretical understanding in current literature, this study aims to clarify the inherent correlation between tempering cycle regulation, microstructural evolution and multi-scale service performance of H13 hot work die steel, and further reveal the essential mechanism of temperature-cycle coupled tempering behavior governing room and high-temperature tribological performance. The prominent novelty of this study lies in breaking through the limitation of existing studies that mostly focus on single room-temperature mechanical performance or conventional isothermal double tempering rules. Different from previous macroscopic performance-oriented investigations, this work concentrates on the differentiated regulation effect of variable-temperature single and double tempering on microstructural characteristics and high-temperature wear behavior. More importantly, this study attempts to clarify the decoupling relationship between traditional hardness-wear resistance cognition and actual high-temperature tribological performance, and clarify the dominant role of surface tribological behavior in controlling the service performance of tempered die steel. The relevant research conclusions can complement and improve the theoretical system of tempering strengthening and wear resistance mechanism of hot work die steel, and provide reliable theoretical support for the performance optimization and engineering application of H13 steel under complex variable-temperature service conditions.

## 2. Materials and Methods

### 2.1. Test Material

**Figure 1:**
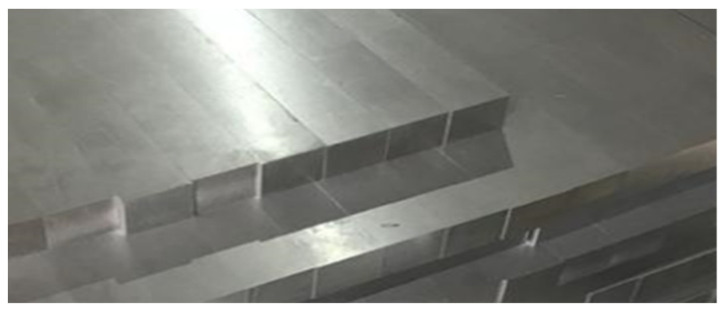
Industrial-grade H13 hot work die steel image.

The material used in this test was an industrial-grade H13 hot work die steel plate with a thickness of 10 mm, processed by hot rolling and in the annealed initial state, as shown in Figure 1.

Its chemical composition was directly provided by Kunshan Tebel Metal Materials Co., Ltd. (Kunshan, China), and the specific composition is shown in Table 1, which complies with the GB/T 1299-2014 standard [8] for alloy tool steels.

### 2.2. Test Equipment and Instruments

The main equipment and instruments used in this test are shown in Table 2, covering all procedures, such as material processing, heat treatment, performance testing, and microstructure observation. All equipment was calibrated to ensure the accuracy and reliability of the test data. Among them, the box-type resistance furnace was used for quenching and tempering H13 steel, which can accurately control the heating temperature and holding time; the optical metallographic microscope and scanning electron microscope were used to observe the metallographic structural characteristics and worn surface morphology of H13 steel after tempering; the microhardness tester was used to test the microhardness of the material; and the reciprocating friction and wear tester was used to test the friction and wear performance under room- and high-temperature conditions, which can simulate the friction conditions during the actual service process of the die.

### 2.3. Test Plan Design

#### 2.3.1. Specimen Processing

The forged and rolled H13 steel plate was processed into standard specimens using a wire-cutting machine for subsequent heat treatment and various performance tests. Among them, the size of the specimen for microstructure observation was 10 mm × 10 mm × 5 mm; the size of the specimen for the microhardness test was the same as that for microstructure observation; and the size of the specimen for the friction and wear test was 25 mm × 25 mm × 5 mm. The surface of the specimen was ground and polished to ensure a surface roughness of Ra ≤ 0.8 μm, so as to avoid the influence of surface defects on the test results for friction and wear performance. After processing, all specimens were cleaned with absolute ethanol, dried, and set aside.

#### 2.3.2. Heat Treatment Process

The heat treatment process of “quenching + tempering” was adopted in this test. All specimens were subjected to the same quenching process, and only the number of tempering cycles and the tempering temperature were changed. It is well documented that conventional double tempering for H13 steel generally adopts identical temperature parameters to eliminate residual stress, whereas the present study adopts differentiated tempering temperature settings for single and double tempering based on practical die service requirements and fundamental material strengthening mechanisms. Specifically, 520 °C is a typical minuteary hardening temperature for H13 steel, which can effectively promote the precipitation of fine carbides and maximize the matrix hardness and wear resistance after single tempering. In comparison, the increased temperature of 580 °C for minuteary tempering is intended to further optimize toughness and thermal stability, which matches the actual variable-temperature heat treatment strategy commonly used for high-performance hot-work dies in industrial production. To clearly distinguish the individual effects of tempering temperature and tempering cycles on microstructure and mechanical properties, the temperature gradient and cycle variables were systematically set in this work, aiming to explore the coupling influence mechanism of tempering parameters on the comprehensive performance of H13 steel. The specific process parameters are as follows:

(1) Quenching process: All specimens were placed into a box-type resistance furnace, slowly heated to 1000 °C, and held for 30 min; after the holding period, the specimens were quickly removed and immersed in room-temperature engine oil to cool to room temperature.

(2) Tempering process: The quenched specimens were divided into two groups, which were subjected to single tempering and double tempering, respectively, with the tempering temperatures set at 520 °C and 580 °C. The specific parameters are as follows:

Single-tempering group: The quenched specimens were placed into a box-type resistance furnace, heated to the set tempering temperature of 520 °C, held for 2 h, and air-cooled to room temperature after the holding period.

Double-tempering group: The specimens after single tempering were heated to the set tempering temperature of 580 °C, held for 2 h, and air-cooled to room temperature after the holding period. Three specimens were selected for each tempering process to ensure the repeatability of the test data.

### 2.4. Test and Analysis Methods

#### 2.4.1. Microstructure Observation

All heat-treated H13 steel samples were cut into uniform size specimens by wire cutting, and surface oil stains, oxide layers and impurities were ultrasonically cleaned with absolute ethanol and dried thoroughly in advance to ensure a clean and flat testing surface. The surface of the heat-treated H13 steel was sequentially ground and polished with metallographic sandpaper, then etched with a 4% nitric acid-ethanol solution for 5–10 s, rinsed with clean water, dried, and set aside. A optical metallographic microscope was used to observe the microstructure of the specimens, and micrographs were taken in different areas of the specimens to analyze the influence of tempering cycles and tempering temperature on the microstructure of H13 steel. A scanning electron microscope was used to observe the microstructural details and carbide morphology of the specimens, so as to further analyze the precipitation characteristics of carbides.

#### 2.4.2. Microhardness Test

Prior to the microhardness test, all specimens were subjected to ultrasonic cleaning with absolute ethanol to remove surface contaminants, followed by natural drying. The tested surfaces were preliminarily leveled to eliminate macroscopic unevenness and ensure parallel contact with the test indenter. All microhardness measurements were performed in accordance with the national standard GB/T 4340.1-2009 [9] using a Vickers microhardness tester to characterize the surface hardness of tempered H13 steel specimens. Before the test, the surface of the specimen was polished until it was free of scratches, the test load was set to 1 kgf, and the load holding time was 10 s. Five different test points were selected for each specimen, and the average value was taken as the final hardness value of the specimen. During the test, surface defects and edge areas were avoided to ensure the reliability of the test results.

#### 2.4.3. Wear Resistance Test

Before the friction and wear test, all specimens were cut into standard test dimensions, ultrasonically cleaned in absolute ethanol to remove surface grease, oxide debris and residual impurities, and completely dried to avoid the interference of surface contaminants on friction and wear test results. A friction and wear tester was used to test the room-temperature and high-temperature friction and wear performance of the specimens, and the test followed the national standard (GB/T 12444.2-2019 [10]). The test temperatures were room temperature (25 °C) and high temperature (580 °C), respectively. Considering that H13 hot work dies bear instantaneous high-pressure contact and local intense friction during actual hot extrusion service, the ball-on-flat contact configuration adopted in this test can effectively simulate the extreme local contact stress state of the die surface in service. In the test, GCR15 steel balls with a diameter of 3 mm were used as abrasives, the motor speed was 560 rpm, the friction radius was 2 mm, the applied load was 1650 g, and the test time was 20 min. The adopted high contact pressure serves as an accelerated screening method, which can rapidly amplify the differences in wear resistance of H13 steel under different tempering treatments and intuitively distinguish the influence of microstructural differences on wear performance. To guarantee the reliability and repeatability of the tribological test results, three replicate tests were conducted for each group of tempered specimens under identical experimental parameters. The friction curves, wear morphologies and overall test phenomena maintained good consistency across all trials.

Before the test, the initial mass of the specimen was weighed with an electronic balance (accuracy 0.001 g); after the test, the wear products on the surface of the specimen were cleaned with absolute ethanol, dried, and weighed again. The wear loss of the specimen was calculated from the mass difference (wear loss = initial mass − mass after test). Although the single test duration was relatively short, this accelerated wear test can effectively avoid thermal fatigue failure and surface oxidation interference caused by excessively long high-temperature testing, ensuring that the test results mainly reflect the friction and wear mechanism dominated by the material microstructure. At the same time, the friction coefficient curve during the test was recorded to analyze the influence of tempering cycles and tempering temperature on the friction coefficient. After the test, a scanning electron microscope was used to observe the worn surface morphology of the specimens and analyze the wear mechanism. Furthermore, the wear mechanism obtained from this accelerated test is mainly abrasive and adhesive wear, which is highly consistent with the typical wear behaviors of H13 dies in practical hot extrusion operations (Ref. [11]). The above comparison fully demonstrates the reliability and engineering significance of the experimental results. The average values of valid test data were adopted for subsequent analysis to eliminate accidental errors and further improve the stability and repeatability of the experimental conclusions.

## 3. Results and Analysis

### 3.1. Effect of Tempering Cycles on the Microstructure of H13 Steel

**Figure 2:**
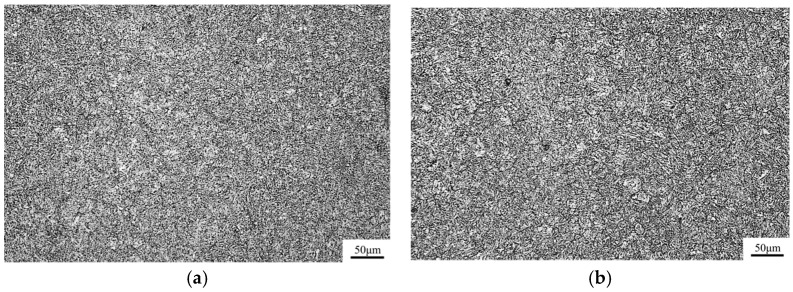
Microstructure photographs of H13 steel after double tempering (**a**) Single Tempering (520 °C) and (**b**) Double Tempering (580 °C).

**Figure 3:**
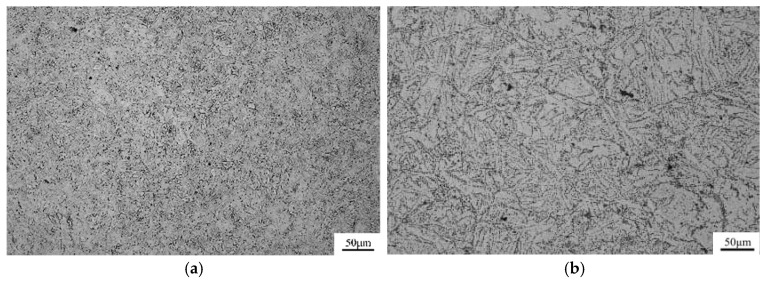
Microstructure photographs of H13 steel after quenching and the base metal (**a**) Quenching (1000 °C) and (**b**) Base metal.

The chemical composition of the selected H13 steel meets the requirements of the GB/T 1299-2014 standard [8] for alloy tool steels. Among them, the C content is 0.40% (within the range of 0.38~0.45%), which can ensure the hardness and strength of the steel; the Cr content is 5.31% (within the range of 4.75~5.50%), which can improve the hardenability and wear resistance of the steel; and the Mo and V contents are 1.10% and 1.04%, respectively (within the ranges of 1.10~1.75% and 0.80~1.20%, respectively), which can significantly improve the tempering stability of the steel and promote the precipitation of fine carbides, thereby enhancing the secondary hardening effect and wear resistance of the steel [12], laying a foundation for the subsequent heat treatment performance optimization and room/high-temperature wear resistance tests. Figure 2 shows the micrographs of H13 steel under different tempering processes, and Figure 3 shows the micrographs of H13 steel after quenching at 1000 °C and of the base metal. It can be seen from Figure 3a that after H13 steel is quenched at 1000 °C, its microstructure is mainly lath martensite with a small amount of retained austenite; the martensite laths are arranged disorderly. It should be clarified that residual internal stress cannot be quantitatively assessed directly from optical micrographs. However, the disordered and irregular arrangement of lath martensite observed in metallographic images is a typical morphological feature induced by high residual quenching stress, which serves as intuitive microscopic evidence for the existence of residual internal stress in quenched H13 steel, and a small amount of undissolved carbides can be observed uniformly distributed in the structure. After the quenched H13 steel is subjected to tempering treatment, the martensite decomposes, fine and dispersed carbides precipitate, the content of retained austenite decreases significantly, and the uniformity of the structure is improved. However, there are obvious differences in the microstructure of H13 steel after single tempering and double tempering. It is acknowledged that the present optical microscopic observation provides qualitative morphological characterization of microstructures and carbide evolution. The carbide types involved in this study are typical precipitated phases of H13 hot-work steel under corresponding tempering conditions, which have been fully verified and universally recognized in extensive previous literature. The morphological differences in carbide size, dispersion degree, and distribution state described in this work are distinct and credible based on high-resolution metallographic images, which can sufficiently support the microstructural evolution analysis in this study. Figure 3b shows the microstructure of the base metal of H13 steel, which is mainly composed of ferrite and spherical carbides.

It can be seen from Figure 2a that after single tempering at 520 °C, the microstructure of H13 steel presents a dense interwoven morphology of dark gray or grayish black. The matrix still retains many lath-like morphological characteristics; although the martensite has decomposed, its directionality remains clearly visible. The extremely small black particles distributed in Figure 2a are the carbides precipitated during the tempering process, and these carbides are in a highly dispersed distribution state. Combined with the mature phase evolution law of H13 steel reported in published studies, these fine, uniformly dispersed nano-scale precipitates formed during low-temperature tempering at 520 °C are typical secondary hardening carbides (Mo_2_C and VC), which are consistent with the classical precipitation characteristics of H13 steel at this temperature. Therefore, the microstructure of H13 steel after single tempering at 520 °C is mainly tempered martensite and finely dispersed carbides. When tempering is carried out at this temperature, H13 steel is prone to secondary hardening. Owing to the fine and dispersed precipitated carbides (such as Mo_2_C and VC), the dispersion strengthening effect can be significantly exerted, enabling the material to maintain high hardness and excellent wear resistance. However, the residual internal stress generated during the quenching process is not completely eliminated.

Compared with Figure 2a, the overall gray scale of the microstructure of H13 steel after double tempering at 580 °C (Figure 2b) is slightly lighter. The extremely fine carbides precipitated after single tempering at 520 °C aggregate and grow, transforming into relatively coarse, bright granular or short rod-like particles; the martensite lath characteristics of the matrix are further blurred, and a flocculent-like structure appears in local areas. In accordance with the classical tempering transformation mechanism of H13 steel, high-temperature secondary tempering promotes the aggregation, growth, and phase transformation of the early precipitated fine carbides. The coarsened large-size particles observed in the microstructure are confirmed as Cr_7_C_3_ and Fe_3_M_3_C carbides, which are the dominant precipitated phases of H13 steel after high-temperature tempering, as widely reported in existing research. At this time, the microstructure of H13 steel is mainly composed of tempered martensite and tempered troostite (or tempered sorbite), and the carbides (such as Cr_7_C_3_ and Fe_3_M_3_C) in the structure are obviously coarsened. After tempering at this temperature, the hardness of H13 steel decreases slightly, but its toughness and plasticity are significantly improved, and the residual internal stress from quenching is fully released.

In summary, after single tempering at 520 °C, the microstructure of H13 steel is mainly tempered martensite and finely dispersed carbides, accompanied by secondary hardening, which can result in high hardness. When H13 steel after single tempering is further subjected to double tempering at 580 °C, its microstructure is transformed into tempered martensite and tempered troostite (or tempered sorbite), the toughness and plasticity are significantly improved, and the residual internal stress is fully released, but the hardness is slightly reduced. The essential reason is that when H13 steel is tempered at a medium-low temperature of 520 °C, fine and highly dispersed special alloy carbides are mainly precipitated, and the dispersion strengthening effect increases the material hardness; that is, the secondary hardening effect occurs. When tempered at a medium-high temperature of 580 °C, the diffusion rate of carbon atoms accelerates, and the alloying elements are redistributed, leading to carbide coarsening and the recovery of martensite laths, resulting in a uniform structure, decreased hardness, and improved toughness.

### 3.2. Effect of Tempering Cycles on the Microhardness of H13 Steel

Table 3 lists the microhardness test results of H13 steel after single tempering and double tempering under different tempering processes. All microhardness measurements were performed on the polished surface of the specimen to ensure flat and uniform test conditions. It can be seen from the data in Table 3 that the microhardness of H13 steel after single tempering at 520 °C is higher than that after double tempering at 580 °C, but the decrease in hardness after double tempering is small, only 1.7% lower than that after single tempering. This test result is basically consistent with the microstructure analysis results mentioned above. In addition, after the two tempering processes, the microhardness of H13 steel is significantly higher than that of its base metal (the hardness of the base metal is 210.35 HV), indicating that tempering treatment can effectively improve the hardness performance of H13 steel.

### 3.3. Effect of Tempering Cycles on the Wear Resistance of H13 Steel

#### 3.3.1. Room-Temperature Wear Resistance

**Figure 4:**
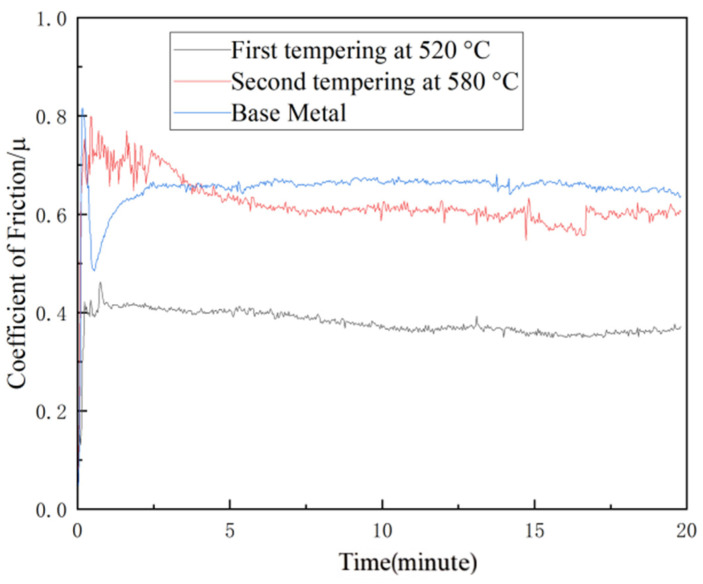
Friction Coefficient Diagram of H13 Steel Samples at Room Temperature (Base Metal, Single Tempering, and Double Tempering).

Figure 4 shows the friction coefficient curves of the H13 steel base metal, the sample tempered once at 520 °C, and the sample tempered twice at 580 °C at room temperature. It can be seen from Figure 4 that the friction coefficient of each sample fluctuates greatly during the initial friction stage (about 1 min), and the friction coefficient tends to stabilize after entering the stable friction stage (after 2 min). Among them, the friction coefficient of the base metal is stable between 0.66 and 0.70, that of the sample tempered once at 520 °C is stable between 0.38 and 0.41, and that of the sample tempered twice at 580 °C is stable between 0.58 and 0.62. Notably, friction coefficient and wear loss are two independent tribological parameters that reflect different material friction and wear characteristics. The friction coefficient mainly characterizes the interfacial friction resistance during the friction process, while the wear loss intuitively reflects the material removal degree after long-term friction and wear. There is no absolute positive or negative correlation between the two indicators, and a low friction coefficient does not strictly correspond to low wear loss, and vice versa.

Table 4 lists the weight loss data for the three samples after the room-temperature friction and wear test. As shown in Table 4, the wear loss of the base metal is 0.00228 g, that of the sample tempered once at 520 °C is 0.00075 g, and that of the sample tempered twice at 580 °C is 0.00141 g. Although the friction coefficient and wear loss of the tested samples show a consistent variation trend in this study, this phenomenon should be regarded as a specific result under the present test conditions rather than a universal rule. This consistency can be attributed to the optimized microstructure and appropriate hardness of the tempered H13 steel, which together improve the interfacial friction state and wear resistance of the material. This result is consistent with the previously discussed microstructural characteristics and microhardness test results, further confirming the influence of the tempering process on the room-temperature friction and wear performance of H13 steel.

**Figure 5:**
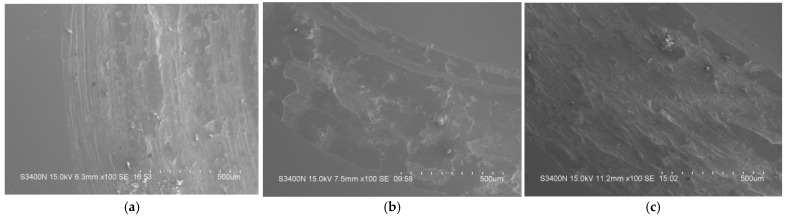
SEM images of the wear scars of H13 steel after the friction and wear test, showing the base metal, single-tempering, and double-tempering samples: (**a**) Base metal, (**b**) Single Tempering and (**c**) Double Tempering.

Figure 5 shows the scanning electron microscope (SEM) images of the wear scars of the base metal, single-tempering, and double-tempering samples of H13 steel after room-temperature friction and wear tests. It is worth clarifying that conventional two-dimensional SEM images cannot provide accurate quantitative depth data for wear grooves. The descriptions of shallow or deep ploughing grooves in this work are relative morphological comparisons based on the planar contour, edge fluctuation, and groove width characteristics of wear scars under the same magnification and test conditions, which are used to qualitatively distinguish the differences in wear degree among different samples rather than characterize absolute depth values. It can be seen from Figure 5b that the worn surface of the single-tempering sample has obvious ploughing grooves, adhesion pits, and wear debris, among which the ploughing grooves are deep and uneven in width, and the adhesion phenomenon is relatively serious, indicating that its wear mechanism is mainly the synergistic effect of ploughing wear, adhesive wear, and abrasive wear. It can be seen from Figure 5c that the ploughing grooves on the worn surface of the double-tempering sample are relatively shallow, but the adhesion phenomenon is still prominent, and its wear mechanism is mainly adhesive wear, which is closely related to the slight decrease in material hardness after double tempering. It can be observed from Figure 5a that, due to the low hardness of the base metal, its wear scar characteristics include those of both the single-tempering and double-tempering samples, specifically manifested as severe ploughing wear, a small amount of adhesive wear, and normal abrasive wear.

#### 3.3.2. High-Temperature Wear Resistance

**Figure 6:**
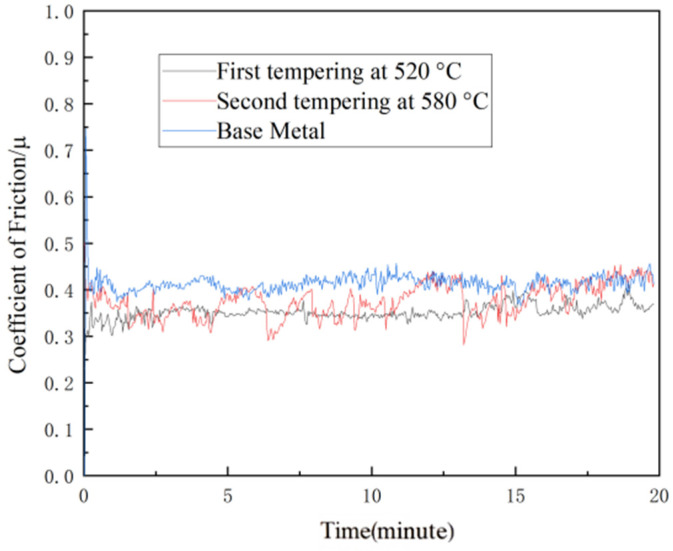
Friction coefficient diagram of H13 steel samples (base metal, after single tempering and double tempering) at the high temperature of 580 °C.

Figure 6 shows the friction coefficient curves of the H13 steel base metal, the sample subjected to single tempering at 520 °C, and the sample subjected to double tempering at 580 °C under the high-temperature condition of 580 °C. It can be seen from Figure 6 that the friction coefficient of each sample fluctuates greatly in the initial stage of friction (within about 0.5 min), and after entering the stable friction stage (after 1 min), the friction coefficient tends to stabilize. Among them, the friction coefficient of the base metal is stable between 0.39 and 0.45, that of the sample subjected to single tempering at 520 °C is stable between 0.35 and 0.40, and that of the sample subjected to double tempering at 580 °C is stable between 0.30 and 0.43.

Table 5 lists the weight loss data of the above three samples after the high-temperature friction and wear test at 580 °C. It can be seen from Table 5 that, under high-temperature wear conditions, the wear loss of the base metal is 0.00436 g, that of the sample subjected to single tempering at 520 °C is 0.00022 g, and that of the sample subjected to double tempering at 580 °C is 0.00034 g. The variation law of the friction coefficient in Figure 6 is basically consistent with the weight loss data in Table 5, further verifying the influence of the tempering process on the high-temperature friction and wear performance of H13 steel.

**Figure 7:**
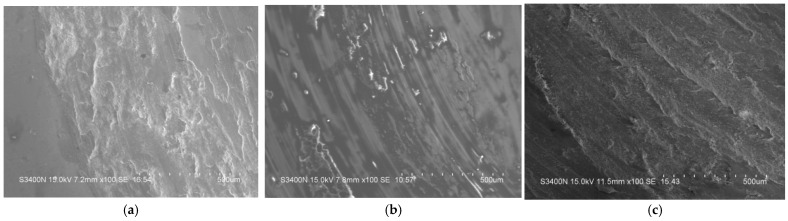
SEM images of the wear scars of H13 steel after friction and wear tests at 580 °C for the base metal, single-tempering, and double-tempering samples: (**a**) Base metal, (**b**) Single Tempering and (**c**) Double Tempering.

Figure 7 shows the scanning electron microscope (SEM) images of the wear scars of the base metal, single-tempering, and double-tempering samples of H13 steel after the high-temperature friction and wear test at 580 °C. It can be seen from Figure 7a that, due to the low hardness of the base metal, its wear scars are mainly dominated by adhesive wear, accompanied by a small amount of ploughing wear. This feature further indicates that the base metal has insufficient hardness and poor wear resistance in high-temperature environments. It can be seen from Figure 7b that the worn surface of the single-tempering sample has obvious ploughing grooves, accompanied by a small amount of adhesive wear and abrasive wear, and the ploughing grooves are shallow, indicating that its surface hardness is relatively high; the overall adhesion phenomenon is relatively serious, indicating that its wear mechanism is the synergistic effect of ploughing wear, adhesive wear, and abrasive wear. It can be observed from Figure 7c that the worn surface of the double-tempering sample is dominated by adhesive wear and deep ploughing wear, which indicates that its surface hardness is lower than that of the single-tempering sample. In addition, it can be clearly observed from Figure 7 that the worn surfaces of all samples exhibit oxidative wear characteristics, which is one of the typical manifestations of high-temperature friction and wear processes.

Comprehensive analysis shows that, under both room-temperature and high-temperature conditions, the wear resistance of H13 steel after single tempering is better than that of the double-tempering sample, and its wear resistance slightly decreases after double tempering. This result is basically consistent with the aforementioned microstructural characteristics and microhardness test results, further confirming the influence of the tempering process on the wear resistance of H13 steel.

## 4. Discussion

### 4.1. Influence Mechanism of Double Tempering on the Microstructure of H13 Steel

After quenching at 1000 °C, H13 steel presents a metastable martensitic microstructure. This quenched structure contains substantial residual internal stress and a certain fraction of retained austenite. Such unstable structural features make quenched H13 steel unsuitable for direct engineering application. Tempering treatment can effectively optimize the microstructure of quenched H13 steel [13]. During tempering, metastable martensite decomposes, and carbides gradually precipitate.

Single tempering at 520 °C initiates the decomposition of metastable martensite in H13 steel. Carbon atoms diffuse out of the martensite matrix and form fine carbides, primarily Mo_2_C and VC. However, with a tempering holding time of only 30 min, the decomposition of metastable martensite is incomplete, and the transformation of retained austenite is insufficient. As a result, only a limited number of carbides precipitate, most of which accumulate at grain boundaries and structural defects. Consequently, the tempered H13 steel exhibits poor microstructural uniformity and a relatively high retained austenite content [14]. Overall, single tempering at 520 °C cannot fully relieve quenching-induced residual internal stresses, which disturb the arrangement of martensite laths and ultimately compromise the microstructural stability of H13 steel [15].

Double tempering at 580 °C is conducted on H13 steel after prior single tempering at 520 °C. On the basis of the tempered martensite and pre-precipitated carbides formed during single tempering, the secondary heating and holding process further decomposes residual metastable martensite. It also promotes the complete transformation of residual retained austenite. The carbides generated during single tempering act as nucleation sites. These sites facilitate the precipitation of additional fine and uniformly dispersed carbides [16]. Double tempering at 580 °C fully releases residual internal stress and eliminates its adverse effects on the microstructure. The martensite laths therefore become finer and more regularly arranged, and carbides are more uniformly distributed throughout the matrix. The overall microstructural stability of H13 steel is significantly improved [17]. In addition, the atomic diffusion rate increases at 580 °C. This phenomenon causes slight coarsening of martensite laths and carbides, which marginally reduces the hardness of H13 steel but greatly enhances its toughness. Compared with single tempering, double tempering at 580 °C more effectively restricts carbide coarsening and reduces the retained austenite content. It therefore achieves a better comprehensive microstructure and optimizes the toughness of H13 steel.

Alloying elements, including Cr, Mo, and V, dominate the microstructural evolution of H13 steel during tempering. Chromium improves the hardenability and tempering stability of the steel and suppresses carbide coarsening. Molybdenum promotes the precipitation of fine Mo_2_C carbides and strengthens the matrix through fine-grain strengthening. Vanadium forms stable VC carbides to enhance hardness and wear resistance and prevent grain coarsening. Double tempering at 580 °C sufficiently activates the functions of Cr, Mo, and V. These alloying elements drive the full precipitation and uniform distribution of carbides, which further optimizes the microstructure of H13 steel [18].

### 4.2. Influence Mechanism of Double Tempering on the Microhardness of H13 Steel

The microhardness of H13 steel is mainly determined by the martensite matrix, the precipitation strengthening effect of carbides, and the content of retained austenite [19]. After H13 steel is quenched at 1000 °C, it can obtain high hardness, but its toughness decreases significantly. When the quenched H13 steel is tempered, the decomposition of martensite leads to a slight decrease in hardness, while the precipitation strengthening effect of internal carbides can effectively compensate for this decrease, resulting in the secondary hardening effect of H13 steel [20].

When H13 steel is subjected to single tempering at 520 °C, the martensite structure in the quenched H13 steel is not completely decomposed, the amount of precipitated carbides is small, and the distribution is uneven, leading to the weakening of the precipitation strengthening effect of carbides. At the same time, the content of retained austenite in the steel remains high, and the hardness of retained austenite is low. Under the combined effect of the above factors, the hardness of quenched H13 steel decreases after single tempering at 520 °C [21]. In addition, single tempering at 520 °C cannot completely release the residual internal stress in the steel. The residual internal stress causes stress concentration in the steel, which further seriously affects the accuracy of the hardness test results and leads to an apparent low surface hardness value.

When H13 steel after single tempering at 520 °C is subjected to double tempering at 580 °C, the incompletely decomposed martensite in the steel is fully decomposed, the carbides are fully precipitated, and their distribution is more dispersed and their size finer, so the precipitation strengthening effect is significantly improved [22]. At the same time, double tempering can significantly reduce the content of retained austenite in the steel, and most of the retained austenite is transformed into martensite with high hardness, further improving the hardness of H13 steel. In addition, double tempering at 580 °C can fully release the residual internal stress of the steel, effectively inhibit the adverse effect of stress concentration on the hardness test results, ensure the accuracy of the test results, and make the apparent hardness close to the actual hardness of the material. Although, during double tempering at 580 °C, the carbides in the steel coarsen and the martensite laths coarsen, leading to weakening of the precipitation strengthening effect and a slight decrease in hardness, the decrease is limited—this is because double tempering can promote the full transformation of retained austenite, and the increase in hardness caused by this transformation compensates to a certain extent for the hardness loss caused by carbide coarsening.

### 4.3. Influence Mechanism of Double Tempering on the Wear Resistance of H13 Steel

Wear resistance is one of the key performance indicators for the service of H13 steel molds, and it mainly depends on the hardness, microstructure, and surface state of H13 steel [23]. It is necessary to clarify that the simple positive correlation between material hardness and wear resistance is only applicable to single pure abrasive wear conditions. For the complex multi-mechanism wear scenario in this study, including ploughing wear, adhesive wear, oxidation wear and secondary debris wear, hardness is no longer the dominant single factor controlling tribological performance, and the traditional hardness-wear resistance correlation cannot be directly applied. The final wear performance is governed by the comprehensive coupling of matrix hardness, microstructure characteristics, tribo-film composition and stability, as well as debris behavior. Notably, tribological properties are surface-dominated behaviors, which are not merely determined by the intrinsic microstructure and mechanical properties of the material. The in situ formed tribo-films (oxide films) on the worn surface during the friction process also exert a critical and direct influence on the friction coefficient and wear behavior, and the synergistic effect of the matrix microstructure and surface tribo-films jointly controls the final tribological performance of H13 steel. Furthermore, the composition of tribo-films and the generation of wear debris are also essential secondary factors affecting the friction and wear behavior. The elemental composition and structural integrity of tribo-films determine their anti-friction and protective capacity, while the quantity, size and morphology of wear debris can change the contact state between friction pairs, thereby aggravating or alleviating abrasive wear.

Under room-temperature conditions, the hardness of H13 steel samples after single tempering at 520 °C is improved, mainly because relatively uniformly distributed carbides are precipitated in the steel during the tempering process. Although there is a small amount of carbide aggregation, the overall distribution is uniform. The higher matrix hardness of the single tempering sample can suppress plastic deformation and reduce adhesive wear tendency to a certain extent, but it cannot independently determine the overall wear performance due to the coexistence of multiple wear mechanisms. Therefore, the friction coefficient of the sample is low, and its wear mechanism is mainly ploughing wear, accompanied by a small amount of adhesive wear and abrasive wear, indicating that the surface hardness of H13 steel exerts a partial optimizing effect on its room-temperature tribological properties at this time. After double tempering at 580 °C, although the structure of H13 steel is more uniform, the carbides are more dispersed, and the size is stable, its surface hardness slightly decreases and the toughness is significantly improved. The increased toughness promotes surface plastic deformation during friction, which intensifies adhesive wear and offsets the beneficial effect of mild structural optimization on wear resistance, rather than simply causing performance deterioration due to reduced hardness. This leads to more obvious adhesive wear, reduced ploughing wear and abrasive wear, and thus a higher friction coefficient than that of the single-tempering sample [24]. Different from high-temperature friction conditions, no dense and stable tribo-film is formed on the worn surface at room temperature. Hence, the room-temperature tribological difference is primarily dominated by the combined effect of the matrix microstructure, hardness, and toughness matching of the two tempered samples. Meanwhile, room-temperature friction produces more irregular hard debris particles, which directly participate in abrasive wear and further expand the tribological differences between different samples.

Under the condition of a high-temperature friction and wear test at 580 °C, the hardness of H13 steel decreases with increasing temperature, and the plasticity increases accordingly. Different from room-temperature friction behavior, high-temperature tribological performance is significantly governed by the growth, integrity, and stability of in situ generated tribo-oxide films on the friction surface, which act as a core factor regulating the surface friction state and wear degree. At high temperature, the influence of matrix hardness is further weakened, and the wear behavior is almost decoupled from the conventional hardness-wear correlation, which is completely different from the single abrasive wear mode dominated by hardness. In terms of tribo-film composition, high-temperature oxidation facilitates the formation of complex iron-based oxide composites on the worn surface. The differences in matrix microstructure and carbide distribution caused by different tempering processes further lead to subtle differences in oxide film composition and compactness, which ultimately affect the surface protection effect. In this environment, an oxide film forms on the worn surface, which can reduce material wear to a certain extent; however, if the oxide film falls off, it will aggravate wear on the material surface [25]. The peeled oxide layers and fractured matrix materials form new abrasive debris, which participates in secondary wear and further changes the wear degree of the material surface. For H13 steel after single tempering at 520 °C, although its tempering stability is reduced, the hardness decrease at high temperature is not obvious, the plastic deformation is small, and the stable matrix microstructure is conducive to the formation of a continuous, dense, and firmly bonded tribo-oxide film that is not easy to fall off. This relatively uniform oxide composition and low debris generation endow the sample with stable interfacial friction characteristics. Therefore, its friction coefficient is better than that of the base metal and the double-tempering sample; at the same time, its friction coefficient is lower than that at room temperature, which is speculated to be related to the protective effect of the oxide film at high temperature.

For H13 steel after double tempering at 580 °C, it has good tempering stability and can maintain a certain hardness and microstructural stability at high temperature [26]. During the high-temperature friction and wear test, a relatively complete and stable oxide film is formed on its surface, but the relatively higher surface toughness and easier plastic deformation of the matrix weaken the bonding strength of the tribo-film, resulting in poorer anti-spalling performance compared with the single-tempering sample. The unstable tribo-film is prone to local peeling, producing more oxide debris. The accumulated abrasive debris on the friction interface aggravates the interfacial fluctuation of friction coefficient and increases material removal. Therefore, its friction coefficient is lower than that of the double-tempering sample at room temperature, but higher than that of the single-tempering sample at high temperature, which further verifies that the high-temperature wear difference is mainly controlled by tribo-film stability and debris behavior, rather than simply determined by matrix hardness.

In addition, the difference in wear mechanism also leads to the difference in wear resistance between the single-tempering and double-tempering samples: the wear mechanism of the single-tempering sample is mainly ploughing wear, accompanied by adhesive wear and abrasive wear; the wear mechanism of the double-tempering sample is mainly adhesive wear. It is worth noting that both single-tempering and double-tempering samples are accompanied by oxidative wear during high-temperature wear, which is one of the typical characteristics of high-temperature friction and wear [25]. Overall, the high-temperature tribological performance of H13 steel is the result of the coupling effect of the intrinsic matrix microstructure and extrinsic surface tribo-film characteristics, rather than a single function of microstructural properties. The stability of tribo-films plays a decisive role in regulating the friction and wear behavior under high-temperature service conditions. Comprehensively, the compositional differences of tribo-films and the generation behavior of wear debris are indispensable auxiliary factors for analyzing tribological performance. In the multi-mechanism wear system of this study, hardness only serves as a secondary influencing factor. The synergistic variation of matrix microstructure, hardness-toughness matching, tribo-film composition and debris behavior jointly determines the final friction and wear properties of H13 steel under different tempering processes, and the traditional single hardness-wear correlation rule is no longer applicable.

### 4.4. Research Limitations and Future Research Directions of H13 Steel

This study has certain limitations: the test selected only two tempering temperatures, 520 °C and 580 °C, focusing on the influence of single tempering at 520 °C and double tempering at 580 °C on the microstructure, microhardness, and room-/high-temperature wear resistance of H13 steel, without considering the regulatory effects of process parameters such as tempering holding time and cooling method on the properties of H13 steel. At the same time, only the rolling friction method was used in the wear resistance test, which failed to simulate the complex load and speed changes during the actual service of H13 steel molds, resulting in a certain gap between the test results and actual engineering application scenarios.

To address the above limitations, future research will be carried out and improved in the following aspects: first, the research range of tempering temperature will be expanded, and the influence of multi-parameter coupling, such as tempering cycles, tempering temperature, tempering holding time, and cooling method, on the properties of H13 steel will be systematically explored; second, the wear resistance test conditions will be optimized to simulate the complex working conditions during the actual service of H13 steel molds, and the tempering process parameters of H13 steel will be further optimized and improved; third, the morphology, size distribution, and evolution characteristics of carbides will be observed by transmission electron microscopy (TEM), the internal relationship between carbide transformation and the properties of H13 steel will be deeply revealed, and more solid theoretical support and technical guidance will be provided for the practical engineering application of H13 steel molds.

## 5. Conclusions

This study investigated the microstructure, hardness, and tribological properties of H13 steel under single tempering at 520 °C and double tempering at 580 °C, and the main conclusions are summarized as follows:

(1) The tempering regime significantly tailors the microstructure of H13 steel. Single tempering at 520 °C produces tempered martensite and finely dispersed carbides with residual internal stress and slight secondary hardening. Double tempering at 580 °C further decomposes martensite, eliminates retained austenite and residual stress, and forms uniform tempered martensite and sorbite structures with improved toughness and plasticity.

(2) Double tempering slightly reduces the hardness of H13 steel. The hardness of the 520 °C single-tempered sample (590.83 HV) is marginally higher than that of the 580 °C double-tempered sample (580.60 HV), with a reduction of only 1.7%. Both tempered samples exhibit much higher hardness than the base metal. This slight hardness difference is governed by carbide precipitation and residual austenite evolution.

(3) Single tempering achieves superior room- and high-temperature wear resistance with a lower friction coefficient and wear loss. The single-tempered sample is dominated by ploughing wear, while the double-tempered sample suffers severe adhesive wear, with oxidative wear occurring at high temperature. Tribological performance is not solely controlled by hardness but is comprehensively determined by the microstructure, hardness-toughness matching, tribo-film stability, and wear debris. Double tempering causes plastic deformation and unstable tribo-films, thereby deteriorating wear resistance.

(4) The two tempering processes are adapted to different service conditions. Single tempering at 520 °C is applicable for high-hardness and wear-resistant molds, whereas double tempering at 580 °C is suitable for impact-resistant molds requiring high toughness. The inferior wear performance of double-tempered samples originates from altered surface tribological behavior rather than minor hardness loss. Double tempering eliminates internal stress and suppresses cracking, extending mold service life. The obtained tempering parameters provide reliable guidance for H13 steel engineering applications.

## Figures and Tables

**Table 1:** Chemical composition of H13 steel (mass fraction, %).

Element	C	Cr	Mo	V	Si	Mn	P	S	Ni	Cu	Fe
Mass fraction (%)	0.40	5.31	1.10	1.04	0.83	0.43	0.013	0.002	0.01	0.01	Balance

**Table 2:** Main Test Equipment and Instruments.

Equipment Name	Manufacturer	Model	Purpose
Box-type Resistance Furnace	China Hefei Kejing Materials Technology Co., Ltd. (Hefei, China)	KSL-1100X	Perform quenching and tempering
optical metallographic microscope	China Suzhou Yueshi Precision Instrument Co., Ltd. (Suzhou, China)	YP710TR	Observe microstructure
Scanning Electron Microscope	Hitachi, Ltd. (Tokyo, Japan)	S3400	Observe morphology of the wear surface
Microhardness Tester	China Laizhou Hengyi Test Instrument Co., Ltd. (Laizhou, China)	WHVS-1000AT	Measure microhardness
Friction and Wear Tester	China Lanzhou Zhongke Kaihua Technology Development Co., Ltd. (Lanzhou, China)	HT-1000	Measure friction and wear properties at room and high temperatures
Wire-cutting Machine	China Suzhou Hanqi Numerical Control Equipment Co., Ltd. (Suzhou, China)	DK7732	Process test samples
Metallographic Polishing Machine	China Jinan Hengsi Shengda Instrument Co., Ltd. (Jinan, China)	MP-2B	Grind and polish samples

**Table 3:** Microhardness Values of H13 Steel with Different Tempering Cycles (HV).

Tempering Cycles	Test 1	Test 2	Test 3	Test 4	Test 5	Mean Value
Single Tempering	565.52	595.56	595.38	598.92	598.79	590.83
Double Tempering	575.12	578.23	575.35	575.21	599.07	580.60

**Table 4:** Weight Loss of H13 Steel After Wear at Room Temperature (Base Metal, Single Tempering, and Double Tempering).

Samples	Before the Test (g)	After the Test (g)	Weight Loss (g)
Base metal	15.13213	15.12985	0.00228
Single Tempering	13.8581	13.85735	0.00075
Double Tempering	13.31299	13.31158	0.00141

**Table 5:** Weight Loss of H13 Steel Base Metal, Single-Tempering, and Double-Tempering Samples After Wear at the High Temperature of 580 °C.

Samples	Before the Test (g)	After the Test (g)	Weight Loss (g)
Base metal	14.60428	14.59992	0.00436
Single Tempering	13.51092	13.5107	0.00022
Double Tempering	13.04851	13.04817	0.00034

## Data Availability

The original contributions presented in this study are included in the article. Further inquiries can be directed to the corresponding author.
